# Analyzing night illumination in dependency of cloud coverage and altitude

**DOI:** 10.1038/s41598-026-68638-9

**Published:** 2026-09-09

**Authors:** Jürgen Krieg

**Affiliations:** Fraunhofer Institute (IOSB) of Optronics, System Technologies and Image Exploitation, Gutleuthausstraße 1, 76275 Ettlingen, Germany

**Keywords:** Climate sciences, Optics and photonics

## Abstract

Despite the publication of a large number of scientific studies in recent years, the impact of clouds on night illumination remains inconclusive. It is also unclear how clouds affect the ever-increasing prevalence of artificial lighting and the subsequent brightening of the night sky. There are many reasons for this uncertainty. Often, the altitude at which the clouds are located is unknown. Furthermore, different measurement equipment is used, which makes it difficult to compare measurements. Additionally, sufficient measurements must be available, as data can vary considerably within a single night and from night to night. To address these issues, Fraunhofer IOSB has conducted measurements in the photopic visual (380–780 nm) and short-wave infrared (800–1700 nm) spectral ranges. These measurements reveal significant variability in terms of cloud coverage and altitude. Comparing these measurements with others has shown that, ultimately, a hemispheric measurement of night illumination combined with the simultaneous determination of cloud coverage and altitude is necessary to draw meaningful conclusions about the influence of artificial lighting on night illumination.

## Introduction

The significant impact of artificial lighting at night (ALAN), also known as light pollution, on our modern environment can no longer be denied. It is now the subject of intense research. An important result of this research shows that the nights are getting brighter in almost all parts of the Earth^1–4^. This increased night illumination has a negative effect on vegetation^5–7^, wildlife^8–11^, and humans^12–14^.

Despite these studies, there is a lack of knowledge to which magnitude the ALAN influences the night illumination. Known factors which influence the night illumination are the lighting installed around the measurement site^15–17^, the aerosol content of the atmosphere^18,19^ and the clouds. For the latter it has been shown that the degree of coverage and altitude are particularly important parameters^20,21^.

In addition to these factors, which are not always easy to determine or measure, the use of different measurement equipment and methods prevents uniform night illumination measurements. For this reason, it is difficult to compare the measurement results.

Fraunhofer IOSB conducted long-term measurements for altogether 14 months between 2019 and 2023 to characterize night illumination in the visible (VIS) and shortwave infrared (SWIR) spectral range for night vision applications. The measurement site was near Storkow in Brandenburg, Germany, a rural site about 50 km southeast of Berlin. There an automated system simultaneously recorded VIS illuminance, SWIR irradiance, and cloud data (coverage and altitude)^22,23^. This publication aims to provide an overview of our measurement results and demonstrate that, in addition to hemispheric measurements, information on cloud coverage and cloud altitude is required to accurately estimate night illumination.

## Results

Besides moonlight and ALAN, clouds have a major impact on the observation of night illumination. Although the measurement data covers a period of around four years, the values are not evenly distributed. The largest number of measurements occurred when cloud coverage was below approximately 10% or above approximately 90%. In both cases, there were always more than 1,500 data points (7-min averages), whereas otherwise there were always fewer than 300. The corresponding data for this can be found in Table 4 in the ‘Methods’ section. The systematic approach of defining cloud coverage in 10% intervals is also described in other published measurements^24^.

**Fig. 1:**
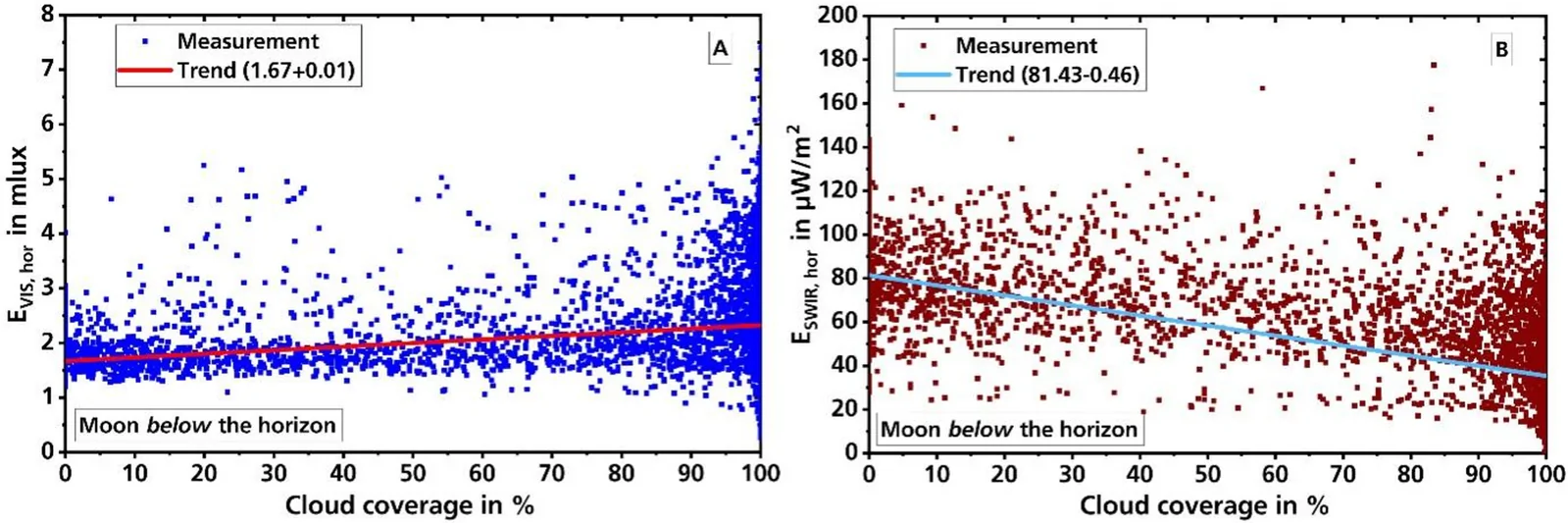
Dependence of night illumination on cloud coverage. Graph A: VIS, graph B: SWIR. The values are 7-minute averages. The lines show the results of linear regressions. Their correlation coefficients are 0.08 (VIS) and 0.45 (SWIR).

Graph A of Fig. 1 plots the measured values of the illuminance meter on cloud coverage when the moon is below the horizon. As shown, the variation is large. It can also be seen that with a cloud coverage of less than 90% the measured illuminance values are never lower than about 1 mlx. Also, there are not many data points above about 4.5 mlx. Only if the cloud coverage increases to about 90% and higher, the measured illuminance values can be found between 0.25 mlx and 7.5 mlx. The red line in Fig. 1A shows the linear regression incorporating all measurements. It starts with an initial value of 1.67 mlx for a cloudless sky and reaches 2.33 mlx for an overcast sky. The calculated slope is then very small, at 0.01 (< 0.01), with standard error in parentheses. See also the values calculated from our measurements in Table 3 in the gray-shaded column.

The linear trends shown in the graphs are intended only to illustrate the general behavior. Based purely on a visual examination of the data, one would expect non-linear behavior, especially in the SWIR. However, due to the uneven distribution of the data (compare Table 4 in the ‘Methods’ section), with many data points at cloud coverage levels below 10% or above 90%, and significantly fewer in between, more measurements are needed to assess whether a linear or non-linear model should be used.

Measurements were carried out simultaneously in both the visible and shortwave infrared spectral ranges. Figure 1B shows the SWIR results when the moon is below the horizon. Again, the large dispersion of the measured values is clearly visible here. When cloud coverage is less than 95%, almost all measured values lie between 20 µW/m² and 140 µW/m². However, when the sky is completely overcast, they can drop to almost zero. The linear regression shows an overall decrease in irradiance with increasing cloud coverage, with an intercept of 81.43 µW/m² (initial value for a cloudless sky) and a slope of −0.46 (0.01), with standard error in parentheses.

Generally, a decrease in illuminance level with increasing cloud coverage would be expected because clouds screen downwelling radiation while reflecting upwelling radiation. Thus, the difference between VIS and SWIR behavior may be explained by the different mixing of natural and artificial lighting. Although artificial light sources may also radiate in the SWIR by emission lines or by temperature radiation caused by heating during operation, this radiation is much smaller than the radiation created in the VIS. In contrast, without the moon, the night illumination in the VIS is known to be roughly one magnitude lower than in the SWIR (compare e.g^25^.).Thus, the high artificial radiation level easily increases the overall illuminance level when reflected by the clouds. For the SWIR the artificial illumination level is too low in contrast to the still transmitted nightglow, and the expected decrease with increasing cloud coverage is observed. Nightglow is a highly variable luminous phenomenon that occurs in the upper layers of the atmosphere (above 80 km) and is mainly found in the SWIR^26^., see Table 1 in^27^.

**Fig. 2:**
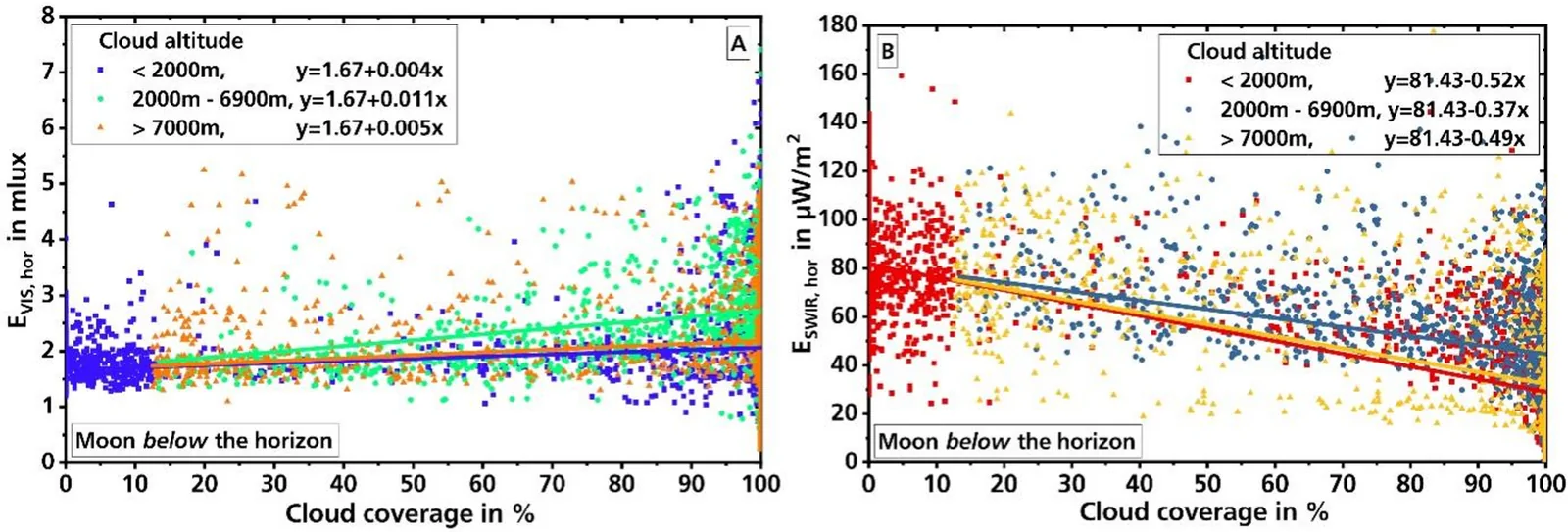
Dependence of night illumination on cloud coverage, divided of three altitude ranges. Graph A: VIS, graph B: SWIR. The values are 7-minute averages. The three lines show the results of linear regressions. The correlation coefficients are (low/medium/high) 0.06, 0.13 and 0.01 for the VIS and 0.58, 0.26, and 0.75 for the SWIR.

As shown in Fig. 2A, in which the altitudes of the VIS measurements are divided into three ranges (see the ‘Method’ section for details), reveals a more differentiated picture. Although all the straight lines calculated from linear regressions start at the same value of 1.67 mlx for a cloudless sky, their slopes vary slightly depending on the altitude range considered. For clouds at an altitude below 2,000 m (the blue line in Fig. 2A), the line shows an increase to 2.1 mlx, while the value for clouds at altitudes above 7,000 m (orange line) rises to 2.2 mlx. The greatest increase is seen in the altitude range of medium-high clouds (2,000–6,900 m, green line), where it reaches 2.7 mlx under overcast skies. This increase therefore contributes most to the overall increase in illuminance shown with the red line on Fig. 1A. One possible assumption for the significant increase in medium-high clouds is that most artificial light sources are located at a sufficient distance from the observation site for the light scattered by the clouds to reach the ground, and thus the measuring instruments.

Our measurements show a clear difference from those of Ribas et al.^20^. They measured a decrease in night illumination compared to clear skies for low and medium-high clouds, and an increase for high clouds (see the ‘Discussion’ section for details). One reason for this could be the differences in artificial lighting around the two measurement sites.

In contrast to the increasing illumination in the VIS, a decrease is observed in the SWIR across all three altitude ranges (Fig. 2B). For low clouds (the red line in Fig. 2B), the value drops to 29.0 µW/m², for medium-high clouds (blue line) to 44.7 µW/m², and for high clouds (yellow line) to 32.3 µW/m². Under clear skies, an average value of 81.4 µW/m² (initial value for all three linear regressions) was measured. This suggests that artificial lighting is the most likely cause of the increase in night illumination in the VIS.

## Discussion

Clouds significantly impact night illumination. Our findings are corroborated by measurements taken at other locations around the world. When these results are categorized by measurement location into three groups - urban, suburban, and rural - clear differences emerge (see Tables 1, 2 and 3). Here, *urban* refers to measurements taken within a major city. The *suburban* category includes measurement sites located in the vicinity of large cities. Measurement sites in the *rural* category, on the other hand, are far removed from large cities. All three tables list the instrument, the measurement site and the night illumination levels for clear and overcast skies. In addition, the difference between the two illumination values was calculated as follows: Difference = (100/E_Clear_ * E_Overcast_) − 100. Where known, the distance to the city center (Table 1) or the nearest major city (Tables 2 and 3) is also provided.

**Table 1:** A selection of clear and overcast sky illumination data collected at *urban* locations. The data had to be converted from sky brightness to illuminance; see the ‘Methods’ section for details. (*): There are multiple measuring locations within the city.

Urban						
**Instrument**	SQM-LU-DL	SQM-L	SQM-LU-DL	SQM-L	SQM-LE	SQM-LE
**Location**	Dalian (CN)^28^	Torun (PL)^29^	Auckland (NZ)^30^	Krakow (PL)^31^	Berlin (GE)^32^	Barcelona (ES)^20^
**E**_**Clear**_	37 mlx	5…9 mlx	8 mlx	25 mlx	8.5 mlx	28 mlx
**E**_**Overcast**_	230 mlx	54…85 mlx	38 mlx	135 mlx	85 mlx	164 mlx
**Difference**	+ 521%	> +500%	+ 375%	+ 440%	+ 903%	+ 486%
**Distance City center**	8 km	(*)	5 km	?	10 km	4 km

**Table 2:** A selection of clear and overcast sky illumination data collected at suburban locations. Some of the data had to be converted from sky brightness to illuminance; see the ‘Methods’ section for details.

Suburban						
**Instrument**	SQM-LE	SQM-LE	SQM-LE	SQM-LU-DL	DSLR camera	DSLR camera
**Location**	Bolechowice (PL)^31^	Potsdam (DE)^33^	Lake Müggel (GE)^32^	Auckland (NZ)^29^	Ludwigsfelde (GE)^34^	Balaguer (ES)^35^
**E**_**Clear**_	2.8 mlx	2.5…5.4 mlx	2.6 mlx	4 mlx	7 mlx	5.6 mlx
**E**_**Overcast**_	19.5 mlx	21.4…85 mlx	11.2 mlx	19 mlx	22 mlx	10.9 mlx
**Difference**	+ 596%	> +296%	+ 331%	+ 375%	+ 214%	+ 95%
**Distance to next major city**	14 km (Krakow)	23 km (Berlin)	18 km (Berlin)	14 km (City Center)	25 km (Berlin)	25 km (Lleida)

**Table 3:**
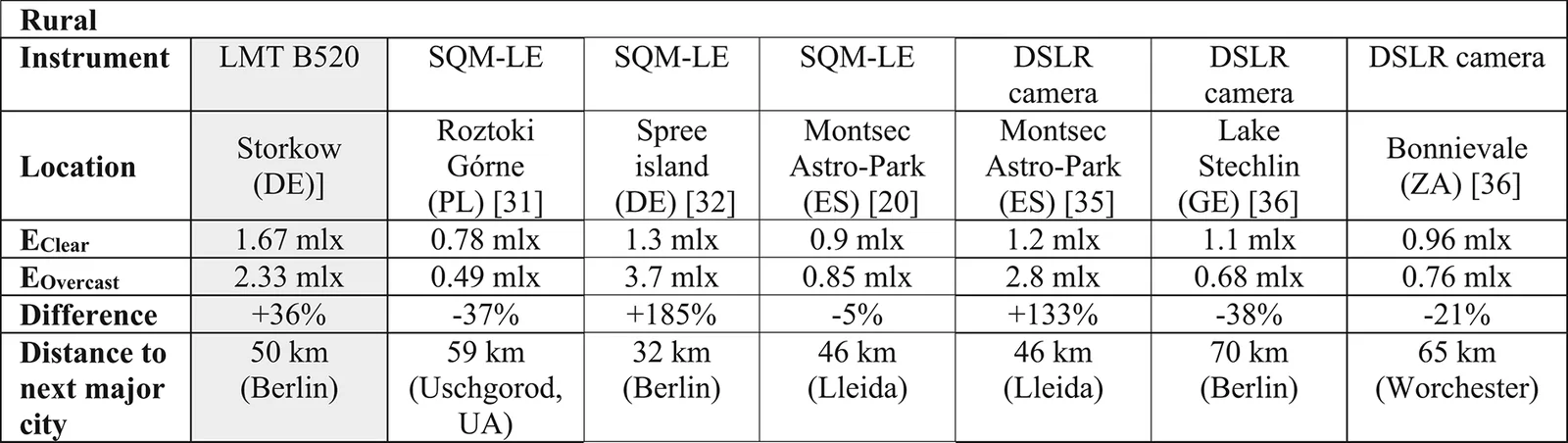
A selection of clear and overcast sky illumination data collected at rural locations. Some of the data had to be converted from sky brightness to illuminance; see the ‘Methods’ section for details. The LMT B520 data are derived from our measurements described in this paper (highlighted in gray).

As shown in Table 1, the data from measurements taken within major cities indicate the expected increase in night illumination under overcast skies. This increase is substantial, typically amounting to several hundred per cent. Even measurements taken in suburban areas (Table 2) show a significant increase in night illumination under overcast skies, although these values are generally lower than those in urban areas. Only in rural locations is the picture more nuanced. While an increase in night illumination under overcast skies was observed at some sites, it decreased at others. The measurement data presented in this paper relate, primarily, to rural locations and can therefore be found in Table 3 (highlighted in gray).

But how meaningful is it to compare data from these different measurements? The underlying assumption that clouds in large cities are illuminated from below by artificial lighting, making the night sky appear brighter, is undisputed. Nor is the assumption questioned that clouds darken the night sky when one is far away from artificial lighting. However, a quantitative comparison of the measurements is problematic, if not impossible. The reasons for this are manifold. On the one hand, the type and number of artificial light sources at and around the measurement site are unknown. On the other hand, different measurement equipment is used. Furthermore, in most cases, only imprecise data on cloud coverage and altitude is available directly at the measurement site. As information on the type and number of artificial light sources is difficult to obtain, only the latter two points will be addressed here.

The most commonly used instrument for measuring the illumination of the night sky is the single-lens version of the Sky Quality Meter (SQM-L). According to the manufacturer, its field of view is 20° (FWHM) with a lens and 84° without, with no cosine correction^37^. Additionally, its spectral response differs from that of the human-eye-adapted illuminance meter used here. During the measurements, the SQM was generally aligned to the zenith^20,32,33^. Also, the measurement units of the instruments (SQM: mag/arcsec², illuminance meter: Lux) cannot easily be converted to a common set of units for comparison since assumptions must be made which are not always entirely accurate (see the ‘Method’ section for details). This complicates the problem and raises the question of how valid the results are when using a small field-of-view for the entire measurement site. Another device used for measurement is a standard DSLR camera fitted with a fisheye lens. This camera can do both measurements – i. e. luminance and illuminance (scalar and horizontal) simultaneously, and the green channel is at least close to the photopic band (closer than the SQM band)^36,38^. The recorded measurement values must still be converted into illuminance values, though^35,39^. The advantage over the SQM lies in its hemispheric coverage of the night sky during measurement. The illuminance meter that we use also performs hemispherical measurements. The result is immediately available in the SI unit Lux (see the ‘Methods’ section for details).

Determining cloud coverage and altitude is a significantly more challenging problem. At night observers can still determine reasonably accurately whether the entire sky is covered with clouds or whether it is clear. However, they cannot provide any information, or can only provide very imprecise information, regarding cloud altitudes. Data from a nearby weather station is usually used to determine cloud coverage and altitude. In such cases, the observation site and the weather station may be several kilometers apart^30,40^. It is then assumed that the cloud conditions are identical at both locations. Some of the measurement results shown in Tables 1, 2 and 3 were taken simultaneously using a ceilometer which was, typically, set up at the measurement site^20,35^. This allows for a fairly accurate determination of cloud altitude. The device’s measurement capacity is limited to the zenith, meaning it cannot be used to determine the degree of cloud coverage. Other methods of obtaining information about cloud coverage and altitude include radar^21^ or image data from DSLR cameras^36^. We also used a Nubiscope as part of our measurements which can determine the degree of cloud coverage and cloud altitude at night (see the ‘Methods’ section for details).

The measurements of Ribas et al.^20^ and Jechow et al.^35^ are a good example of how difficult it is to compare data from two measurement campaigns. Both studies were conducted at the Observatori Astronomic del Montsec in Spain. In addition to differing weather conditions, they also used different measurement equipment. While Ribas et al. found a slight decrease in night illumination under cloud coverage compared to clear nights, Jechow et al. observed an increase in illumination (see Table 3, columns labelled ‘Montsec Astro-Park’). In their paper, however, Ribas et al. distinguish between low, medium-high and high clouds. They measure a decrease in night illumination under low and medium-altitude clouds (0.64 mlx and 0.85 mlx, respectively) but also find that illumination increases to 1 mlx under high clouds (clear sky: 0.9 mlx). Jechow et al. state that the ceilometer measured cloud altitudes of around 6 km which corresponds to mid-level clouds. Therefore, the results of Ribas et al. and Jechow et al. do not match. It is not possible to determine whether the cause lies in the cloud coverage, the measuring equipment, or both. Nevertheless, they emphasize the difficulties associated with such comparisons.

It is also impossible to determine whether the two measurements taken with DSLR cameras in Table 2 show the smallest increase between clear and overcast skies because they were taken farthest from the nearest major city or because entirely different measurement equipment was used. All the other measurements listed in Table 2 were conducted using SQM variants.

When comparing different measurements, the number of data points must also be considered. Sticking with the examples from Ribas et al. and Jechow et al. discussed above, the analyses in those studies were based on very different amounts of data. While Ribas et al. used approximately 66,500 measurements collected over six months, Jechow et al. used data from just two nights. However, as Fig. 1 shows, there is significant variation in the data, and the illumination values taken on a single night with an overcast sky can vary greatly.

All the limitations identified for the other locations naturally also apply for the Storkow data presented here. All measurements are limited by the number of measurements, the measurement equipment and the measurement site. The number of measurements is generally the one to overcome easiest. But even the relatively long measurement duration used showed limitations for analyses, e.g. the uneven distribution of data points with cloud coverage. The measurement devices used here are typical equipment for illumination characterization. However, they have not yet been used elsewhere, nor have they been compared with the most common SQM and DSLR camera equipment at a common measurement site. Last but not least, there is nowadays no practical measurement site free from urban lighting influence. Such a site would be needed to provide a baseline for quantifying the effect of urban lighting. It was here tried to overcome this limitation with the help of the measurement in a different spectral range. Due to the characteristics of artificial light sources, it can be assumed, though not proven, that the SWIR spectral range is unaffected, or at least less affected, by urban lighting.

## Conclusion

Illumination levels measured at night depend heavily on whether the sky is clear or overcast. The measurement location and cloud altitude also influence the results. In urban areas, clouds generally cause the illumination of the night sky to increase, whereas in rural areas it decreases. Taking cloud altitude into account reveals that illumination can increase or decrease depending on the altitude of the clouds. Comparing measurements from different locations is difficult. This is primarily because a wide variety of measurement equipment has been used to date. Furthermore, information on cloud coverage, and especially on cloud altitudes, is often insufficient.

Comparing measurements from different locations is complicated by the fact that significant variability in night illumination can be observed even at a single location. Our data clearly demonstrate this. Therefore, it is important not to rely solely on measurements from just a few nights, but to collect data over as long a period as possible - at least several months, or preferably a year or more. Only in this way, the relevant ranges for one’s own measurement site can be statistically determined for night illumination.

To mitigate the problems described, it would be beneficial to use the same measurement equipment, or at least comparable equipment, over an extended period. Information on cloud coverage and altitude at the measurement site should also be available. However, securing the necessary financial and human resources poses a challenge. Nevertheless, the benefit of being able to compare data on a global scale would be significant. This would allow us to collect data sets that are less open to interpretation and therefore more reliable.

## Methods

Much of the information in this section has already been published in^23^, as the same measurement data was used for that publication too.

### Measurement area

Selecting a suitable measurement area was a difficult process as our application required remoteness from artificial lighting, ease of access and security. These factors exclude each other to some extent: No artificial lighting typically means remote areas that can be difficult to reach and have poor security. Additionally, financial limitations had to be considered and so the search was limited to areas in Germany or Central Europe. The final compromise was a proving ground near Storkow, Brandenburg, Germany (52°11’ N, 13°56’ E, altitude 49 m). The small town is about 8 km away. Although located about 50 km southeast of Berlin, this area is in one of the darkest German regions. The light pollution map^41,42^ provides the following zenith sky brightness values: Proving Ground: 21.6 mag/arcsec^2^ (0.247 mcd/m²), Storkow: 21.02 mag/arcsec^2^ (0.422 mcd/m²), Berlin (Brandenburger Tor): 18.10 mag/arcsec^2^ (6.2 mcd/m²).

A forest clearing sufficiently large to avoid interference with the sensors from shadowing (such as that caused by trees in moonlight) was selected as the measuring area. Around the clearing, there is no artificial lighting at a radius of at least 1.5 km.

### Illumination measurement

**Fig. 3:**
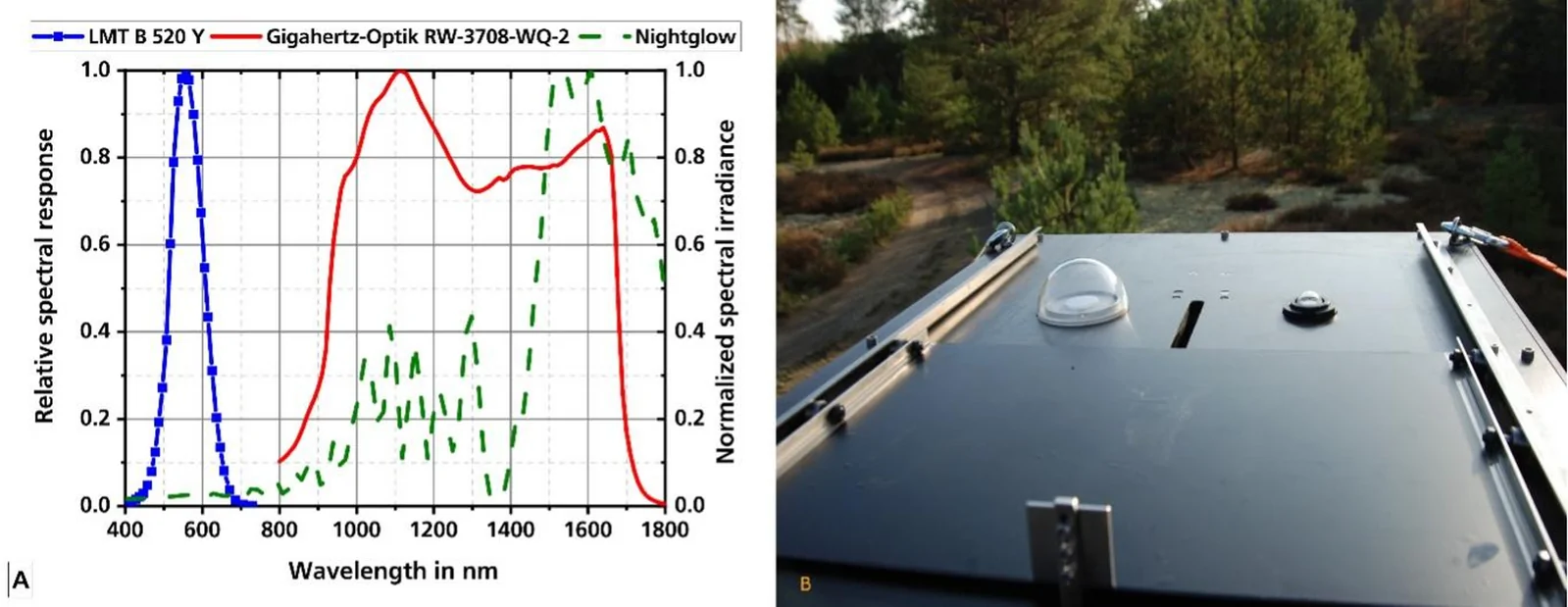
A: Illuminance and irradiance meter relative spectral response in comparison to normalized starlight nightglow spectral irradiance^43^. B: The picture shows the housing with the two sensors under the glass domes. The sliding hood can be seen in the foreground. (Reprinted with permission from^22^© The Optical Society).

The main measurement equipment are an LMT B520 illuminance and a Gigahertz-Optik RW-3708-WQ-2/P-9710-1 irradiance meter. The LMT B520 is a photopic response adapted, cosine corrected device with a minimum resolution of 10 µlx and 2.5 measurements per second with total error given as less than 7.5% by the manufacturer. The spectral response of the LMT B520 in comparison to the normalized starlight nightglow spectral irradiance is shown in Fig. 3A. The irradiance meter consists of the RW-3708-WQ-2 sensor head and the P-9710-1 display unit. It is also cosine corrected, with typical sensitivity of 40 nA/W/m² and recording approximately two samples per second. The ± 7% device uncertainty is similar in magnitude to that of the illuminance meter. Spectral response is broadband, covering the typical SWIR-sensor spectral range from approximately 800 nm to 1700 nm (Fig. 3A). Both sensors are protected by a glass dome attached by the manufacturer. They are calibrated at least biyearly by the manufacturers with calibrations traceable to PTB, the national metrology institute of Germany.

The sensors are mounted in a common housing with the sensitive areas parallel to the ground (Fig. 3B). As a result of the measurements, horizontal illuminance and horizontal irradiance are obtained. An automated sliding hood protects the sensors during the day. This setup is located approximately two meters above ground level to avoid interference from grass and bushes as well as to provide protection from animals.

### Cloud characterization

It is difficult to observe and measure clouds during the night. An IMK Nubiscope^24,44^ based on a scanning pyrometer is used here. The instrument measures cloud coverage in different altitude regions (low: < 2000 m, medium: 2000–6900 m and high: > 7000 m) and also the lowest clouds within 50 degrees around the zenith region (LowCl). It calculates the altitude of clouds in these three regions and the LowCl. The pyrometer’s detector can operate both day and night, automatically scanning the entire sky at ten-minute intervals, with each scan taking approximately seven minutes. Default settings are used for this measurement. LowCl was used for the analysis here.

### Measurement duration

Measurements are not made from mid-May to the end of July because at Storkow latitude astronomical twilight (sun 18 degrees below the horizon) lasts the entire night with sunlight still illuminating the upper parts of the atmosphere^45^. These breaks are used for cleaning, recalibration, etc. For the analysis, data from the following months are available: 2019: March (21.−31.), April, May (1.−19.), August, September, October (1.−20.); 2020: August (4.−31.), September, October (1.−23.); 2023: March, April, May (1.−18.), August, October (18.−30.).

### Data recording

All instruments are connected to a common PC for control and data storage with a remote data download option. The whole system is automated and autonomous. The illumination sensors and Nubiscope operate only during night-time (a few hours before astronomic dawn to a few hours after astronomic dusk). Solar panels and a fuel cell are used to recharge the batteries to provide power, which provides flexibility in choice of location.

### Data processing

The collected illumination data is pre-processed by averaging one-minute intervals. The resulting illumination plots for each night were visually inspected for data collection problems, anomalous illumination effects, etc. Such data is excluded from the analysis. For analysis in connection with cloud data from the Nubiscope, the illumination data is averaged over 7-minute intervals, the approximate time it takes the Nubiscope to scan the sky. The illuminance data is restricted to times between the end (of the previous day) and before the beginning (of the current day) of astronomical twilight. To exclude the influence of moonlight on the illumination level, only times where the moon was below the horizon (< −5 deg.) are considered here.

To investigate the influence of cloud altitudes on the illumination level, the measurements were divided into three altitude ranges: Cloud altitudes lower than 2000 m, higher 2000 m up to 6900 m, higher 7000 m. These altitude ranges are based on the specifications of the German Weather Service (DWD)^46^. To provide an overview of the amount of available data, Table 4 presents the number of measurements divided into 10% cloud coverage intervals. During the observation periods, no medium-high or high clouds were observed when cloud coverage was less than about 12%. The mean value obtained from the data collected under clear skies was used as the initial value for the linear regressions within each spectral range.

**Table 4:** Distribution of the available number of measurements (7-minute-averages) in 10% cloud coverage intervals. Data is given absolute and differentiated for the three altitude ranges defined in the text.

Cloud coverage [%]	0 - <10	10 - <20	20 - <30	30 - <40	40 - <50	50 - <60	60 - <70	70 - <80	80 - <90	90– 100
**All altitudes**	1534	201	131	137	115	109	134	175	251	2319
**Low clouds**	1534	81	15	23	11	19	27	53	98	946
**Medium-high clouds**	0	25	47	62	58	61	77	83	102	752
**High Clouds**	0	96	69	52	46	29	30	39	51	621

### Sky brightness conversion

Illuminance and irradiance are standard figures of merit to characterize illumination. To characterize brightness, luminance is used. Unfortunately, the majority of published data on light pollution and corresponding topics does not include illumination data but ‘sky brightness’. This sky brightness is typically either given in cd/m² or magnitudes per arc seconds square (mag/arcsec²). To compare such data to the one presented here, conversion is necessary. To do so, sky brightness [mag/arcsec²] is transferred first to luminance L [cd/m²] according to the conversion given by Hänel et al^39^.. Assuming that sky brightness is uniform across the sky (which is not necessarily true when clouds are present), luminance and illuminance E [mlx] are related by the factor π. The factor represents a lower limit for the ratio of horizontal illuminance to zenith brightness^47^. This results in the overall conversion function used here whenever needed.

$$\:E\approx\:\pi\:\cdot\:10.8\cdot\:{10}^{4}\cdot\:{10}^{-0.4\cdot\:sky\:brightness}$$

### Software

For the pre-processing IDL 8.4.1 (Interactive Data Language; https://www.nv5geospatialsoftware.com/Products/IDL#language) was used. Plotting and statistical analyses were performed using OriginPro 2026 (https://www.originlab.com/).

## Data Availability

The data sets generated and analyzed during the current study are not publicly available, but are available from the corresponding author on reasonable request.
